# Patch-Based Segmentation with Spatial Consistency: Application to MS Lesions in Brain MRI

**DOI:** 10.1155/2016/7952541

**Published:** 2016-01-24

**Authors:** Roey Mechrez, Jacob Goldberger, Hayit Greenspan

**Affiliations:** ^1^Biomedical Engineering Department, Tel-Aviv University, 69978 Tel Aviv, Israel; ^2^Engineering Faculty, Bar-Ilan University, 52900 Ramat Gan, Israel

## Abstract

This paper presents an automatic lesion segmentation method based on similarities between multichannel patches. A patch database is built using training images for which the label maps are known. For each patch in the testing image, *k* similar patches are retrieved from the database. The matching labels for these *k* patches are then combined to produce an initial segmentation map for the test case. Finally an iterative patch-based label refinement process based on the initial segmentation map is performed to ensure the spatial consistency of the detected lesions. The method was evaluated in experiments on multiple sclerosis (MS) lesion segmentation in magnetic resonance images (MRI) of the brain. An evaluation was done for each image in the MICCAI 2008 MS lesion segmentation challenge. Results are shown to compete with the state of the art in the challenge. We conclude that the proposed algorithm for segmentation of lesions provides a promising new approach for local segmentation and global detection in medical images.

## 1. Introduction

Patch-based methods have been shown to be an effective approach for labeling brain structures (and other body structures), as shown, for example, in [1, 2]. In general, these approaches label each voxel of a target image by comparing the image patch, centered on the voxel with patches from an atlas library, and assigning the most probable label according to the closest matches. Often, a localized search window centered around the target voxel is used. Various patch-based label fusion procedures have been proposed and were shown to produce accurate and robust segmentation. Using affine registration, comparable results to works that use nonrigid registration have been reported. Patch-based techniques have recently demonstrated high performance in various computer vision tasks, including texture synthesis [3], inpainting [4], and super resolution [5]. Nonlocal means denoising [6] has helped advance the field and has led to the development of various patch-based segmentation tools for medical imaging applications methods [1, 2, 7, 8].

The focus of segmentation tasks in existing works is on regions of interest which are substantial in size and/or are anatomically localized (e.g., brain tumors, knee, brain tissues, the hippocampus, cortical parcellation, and brain structures). In these cases the anatomical context provides labeling support and a good approximate alignment of the image to an atlas (expert priors) is needed and is a key component. For example, in the hippocampus or the knee, the algorithm is designed to differentiate between the surrounding tissues and the convex target region; thus the inner voxels are easy to label. Similarly, in the case of brain tissues, anatomical constraints facilitate labeling most of the voxels, and the errors are mainly in the border voxels.

**Figure 1:**
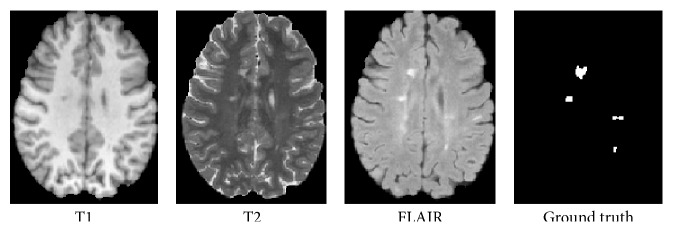
Three axial slices of MS lesions on MRI: FLAIR, T1w, T2w, and the associated ground truth lesion map.

**Figure 2:**
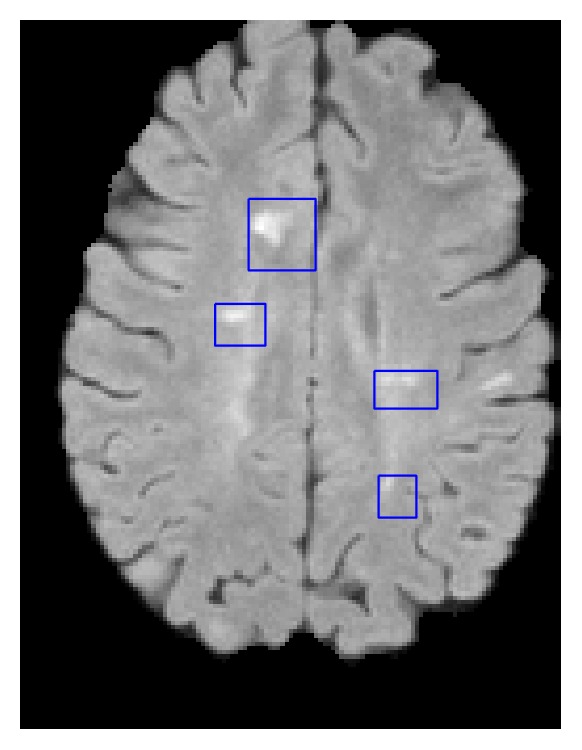
ROI (blue) on a FLAIR image.

**Figure 3:**
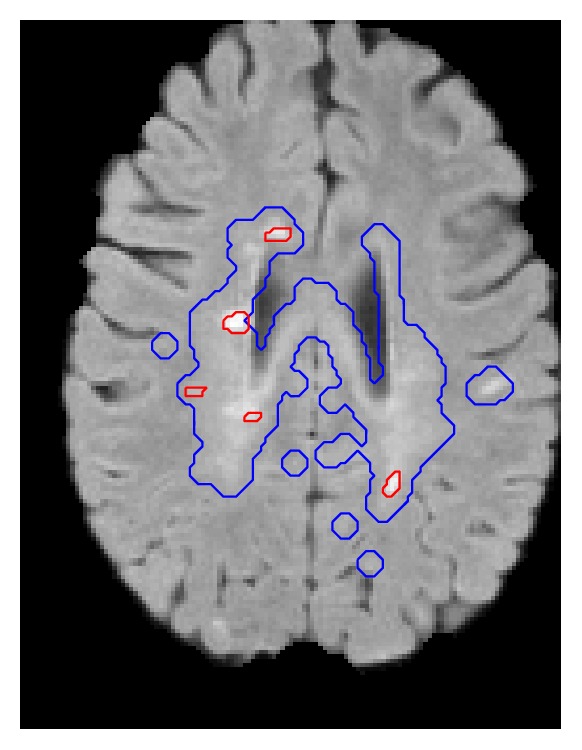
Candidate lesion regions mask (blue) and the lesions (red) on a FLAIR image.

In the current work, we focus on developing a patch-based segmentation for small nonlocalized regions. The regions of interest examined here, such as lesions, do not entail anatomical constraints, and hence the location of these lesions is subject to inconsistency in terms of both neighboring tissues and the absolute position. These regions are not contained within other regions, as is the case for brain tumor or the knee. We aim to overcome the lack of spatial constraints and anatomical context by a novel spatial consistency step, as discussed in Section 2.6. We deal with MS lesion detection and segmentation in MR images of the brain which involve a number of challenges; in particular MS lesions are very small, they can appear anywhere in the brain image, and they are relatively similar to other brain tissues. Examples of MS lesions are presented in Figures 1, 2, and 3.

MS is the most common nontraumatic neurological disease in young adults. It is an inflammatory demyelinating disease that is primarily associated with axonal loss and formation of lesions in the central nervous system, which are characterized by demyelination, axonal injury, and axonal conduction block. These classically described white matter (WM) lesions are visible in conventional magnetic resonance imaging (MRI), appearing as hyperintense in T2-weighted (T2w) images and as hypointense in T1-weighted (T1w) images (see Figure 1). Fluid attenuated inversion recovery (FLAIR) images have been shown to be the most sensitive to WM lesions but can also present other hyperintensity artifacts [9].

MRI is currently used to diagnose of MS, assess disease progression, and evaluate the efficiency of drug therapy [10]. The most common quantitative parameter is the lesion load of the disease, expressed in terms of the number and volume of brain lesions. The MRI measured lesion load is highly correlated with clinical findings [11, 12]. An accurate segmentation at the voxels level is a necessary step to calculate the lesion load or any other measure.

Manual segmentation of WM lesions is a time-consuming process. Furthermore, the 3D data of an MRI scan requires multislice segmentation which makes the manual segmentation a laborious task [13]. Fully automated algorithms for MS lesion detection and segmentation have been the focus of research for many years. Algorithms to date have failed to respond to the complexity of the task and new approaches have recently been introduced [14–17].

Pattern recognition and machine learning techniques have been widely investigated to identify the patterns of MS lesions by making use of the neuroimaging data [18–21]. These segmentation methods can be classified as supervised or unsupervised as a function of the mathematical algorithms they implement.

Unsupervised methods [23, 24] are designed to solve the segmentation task without needing labeled training data. These methods attempt to formalize the definition of a lesion and differentiate lesions from other tissues on the basis of anatomical knowledge. For example, in [21] the MS lesions were identified as outlier from a constrained Gaussian mixture model (CGMM). Likewise, in [16], the lesions are identified as outliers using dictionary learning and sparse coding. Some works [15, 25] use an anatomical atlas as the prior anatomical knowledge, but since these works required accurate 3D registration step, this step may be misdirected in the presence of lesions.

Supervised methods are a useful way to recognize a pattern in a new test sample based on information learned from the training samples. Typically the source of the training labels is manual segmentation. Typically studies use one of the many available supervised learning methods, for example, *k*-nearest neighbor (*k*-NN) [19], support vector machine (SVM) [26, 27], cellular neural networks (CNN) [28], or a Bayesian framework [29]. For example, one of the outstanding works [14] used a discriminative random decision forest to provide a voxel-wise probabilistic classification of the volume.

Since the detection of white matter lesions (WML) is a challenging task, extraction of discriminative features plays an important role in lesion segmentation. Voxel-related features, such as the probabilities of different tissues (gray matter (GM), white matter (WM), and cerebrospinal fluid (CSF)) and the multichannel intensity (i.e., T1, T2, and FLAIR), play an important role in neuroimaging studies. Crucially, although some authors use only one sequence (T1 or FLAIR) [15, 30], the lesion should be confirmed in other sequences to avoid false positives (FPs) [13]. Nevertheless, voxel-related features are not sufficient for good segmentation, since other tissues have a similar appearance, and therefore, spatial information is necessary [13]. Specifically, the spatial information is usually included in the local mean, using the neighborhood information as in many imaging applications [1, 2, 5, 19]. The local spatial information reduces the impact of noise and improves coherence of the results. To use the spatial information, mathematical techniques as Markov random fields (MRF), graph cut, and kernel features are employed [31].

In this study we propose a patch-based method for detection and segmentation of MS lesions in brain MRI by utilizing multimodal spatial information. The segmentation is first obtained based on intensity patch similarity and then further iteratively refined with the spatial label information. The proposed framework segments the MS image without requiring registration or an atlas. This makes the framework robust to registration errors which are likely to occur if there is a high degree of anatomical variability.

The main contributions of this paper can be summarized as follows:

1. Adding a spatial consistency refinement step to the patch-based approach using a novel label propagation based metric.
2. Creating a patch database for the MS task. In MS, the lesion anatomical positions differ significantly between subjects. We therefore cannot use the same anatomical volumes of interest as in classic patch-based segmentation.
3. Providing a framework for segmentation of MS lesions that does not require registration, with no need for an atlas.

The rest of this paper is organized as follows. The proposed method is described in the next section. Then, in Section 3, extensive experiments and comparisons with other segmentation methods on the MICCAI08 grand challenge datasets are presented to demonstrate the segmentation accuracy of the proposed method. Finally, in Section 4, we discuss parameters influences on the proposed framework and possible future directions.

## 2. Patch-Based Segmentation and Label Refinement

### 2.1. System Overview

Our method is based on labeling the test image voxels (as lesion or nonlesion) by finding similar patches in a database of manually labeled images. The training step involves constructing a patch database using expert-marked lesion regions which provide voxel-level labeling. Given a test image, we first detect candidate lesion regions for further analysis. For all candidate voxels we find the *k*-nearest neighbor patches from the patch database. Labels of the selected patches are used to determine the current patch label, using a voting scheme, thus generating the test image label map. Finally, to enforce spatial consistency, we iteratively incorporate a label decision from the neighboring voxels obtained during the previous iteration, by adding them to the *k*-NN metric. Before we applied our method preprocessing steps, subject selection step and candidate region detection are done. In the following subsections we provide a detailed description of the algorithm.

### 2.2. Preprocessing

Before starting the segmentation process (in the training phase as well as for a new test image), a few preprocessing steps are taken. During these steps, variability caused by image formation is minimized by inhomogeneity correction and intersubject intensity normalization. The brain is extracted to avoid nonrelevant tissues (i.e., the skull and scalp).

#### 2.2.1. Subsampling

We subsample the images so that they all are the same size 169 × 129 × 130 and the same isotropic resolution 1 × 1 × 1 mm^3^. The subsampling is designed to reduce the computational time and the background as much as possible. From this step on, voxels outside the brain mask are ignored.

#### 2.2.2. Normalization

The most crucial step in the preprocessing is the normalization of the intensities in the image. This is mandatory to ensure that all brain tissues in different subjects have the same contrast and luminance. We use the normalization procedure of Nyúl et al. [32] to make patch matching between different subjects possible. Some authors use normalization to zero mean and unit standard deviation. In the datasets we experimented with we found that using the intensity as is provided better results. Note that for the analysis we need very good intensity normalizations across brains. In order to support the normalization procedure we add an additional step of subject selection (Section 2.3.3) to select the most similar brains in the training set.

#### 2.2.3. Inhomogeneity Correction

To ensure that each tissue type has the same intensity within a single image, the well-known N4ITK intensity nonuniformity correction in [33] is used on all three channels.

#### 2.2.4. Brain Extraction and Tissue Classification

A brain mask was created using the BrainSuite13 toolbox [34] as described in [35]. Classification to WM, GM, and CSF was also applied using [36]. In this classification process, voxels in an intensity-normalized image are classified using a maximum a posteriori (MAP) classifier. This classifier combines the partial volume tissue measurement model with a Gibbs prior that models the spatial properties of the brain.

### 2.3. Creation of a Labeled Patch Database

Recent patch-based segmentation works are based on the nonlocal means (NLM) idea [6, 37], where similar patches are searched in a cubic region around the location under study. A patch-to-patch similarity in specific anatomical regions is assumed to hold true and the segmentation tasks are considered to have spatial consistency (e.g., the hippocampus in different brains appears in the same region). MS lesions, however, can be found in different brain regions and local region patch matching cannot be implemented. To overcome this shortcoming, we suggest a labeled patch database creation step.

#### 2.3.1. Patch Sources

In the training step, we extract regions-of-interest (ROIs) around all manually marked lesions. A ROI is defined as the bounding box of the lesion (see Figure 2). Note that we expand the borders of the bounding box by a few voxels (three) in order to capture the surrounding tissue as well. ROIs therefore represent the lesion and nonlesion around the lesions in a roughly balanced way. To better represent the entire brain we add randomly selected patches from all brain regions. This way we represent all brain tissues but give special attention to complex lesion regions and the “normal appearing white matter.” From each ROI, and for each input channel, we then extract 3 × 3 × 3 patches as in [16, 38].

#### 2.3.2. Augmented Patch

Patches are concatenated across the input channels (in our case: T1, T2, and FLAIR) to form augmented patches *P* ∈ *R* ^81^ (referred to hereafter as concatenated patches). This set of patches forms the training patch database **D**. We further examine the use of different weights for the three modalities using multiplication by three constants; see Section 2.5.3 for details.

#### 2.3.3. Subject Selection and Patch Selection

In many segmentations tasks, multiple training subjects (or atlases) are used and a process of label fusion is needed to combine the different segmentations. A subject selection step is needed in order to ensure fusion of information from most similar sources. For example, in [1, 39, 40], the nearest subjects are selected for each region that requires labeling, by comparing the Euclidean distance between corresponding regions. In contrast, in our method, we do not use the concept of similar anatomical regions for patch retrieval; rather we use the labeled patch database originating from multibrain regions. We define a* global* subject selection step, to facilitate a selection of similar brains, to increase the accuracy of similarity comparisons between patches. Given a set of *M* training images {*T*_*n*_∣*n* = 1,…, *M*} we select a subset of *N* training images {*T*_*n*_∣*n* = 1,…, *N*} whose Kullback-Leibler (KL) divergence [41] from the test image is minimal. Let *R* and *Q* be the multimodal histograms (i.e., three histograms, one for each modality, concatenated) of the given test image and a train image, respectively. The KL divergence is defined to be

(1) $$\begin{array}{c} D_{\text{KL}}\left(\;R||Q\;\right)=\underset{i}{\sum }R_{i}\cdot \log\frac{R_{i}}{Q_{i}}. \end{array}$$

The subject selection leads to a labeled patch subset *D*_*I*_ ⊂ *D* denoted by *D*_*I*_ = {*P* ^*D*^ ∈ *T*_*n*_∣*n* = 1,…, *N*}. For the training images the label maps {*L*_*n*_∣*n* = 1,…, *N*} are known. Following the subject selection step we select a subset of patches *D*_*I*_′, out of *D*_*I*_, by subsampling in fixed increments, such that the number of lesion and nonlesion patches is equal and the total number of patches reaches a fixed number. The subsampling is done using the index numbers of the extracted patches. Thus the subsampling is in a sense in space; recall that patches from the same ROI have close indexes. From our empirical experimentation we found an appropriate number to be between 100 and 150 thousand patches.

### 2.4. Detection of Candidate Lesion Regions

Next, we focus on automatically detecting candidate lesion regions in a given test image. The detection is based on two clinical rules:

(i) The lesions appear as hyperintense in FLAIR images; thus they can be roughly identified using thresholding. It is well known that the FLAIR input channel is a good source for analyzing MS lesions [13]. Using a global threshold (TH) on a FLAIR image provides high sensitivity in the detection. Since such a threshold results in poor specificity, we can use the FLAIR input to provide an initial rough delineation of candidate lesion regions (see [26] for a detailed description).
(ii) The lesions are characterized by demyelination; thus they are part of the WM tissue. Lesions tend to be found in the WM or on the border between WM and GM. We classify the brain tissues using a maximum a posteriori probability classifier (MAP) [34] (see preprocessing, Section 2.2.4) and extracted the WM region (dilated) as a second mask. The dilation step is done in order to guarantee that lesions surrounded by WM, WM boundaries, and peripheral lesions will be in the mask. The structure element used is a “ball” with 10 pixels radius.

The final set of lesion candidate regions consists of all the voxels above the FLAIR TH intensity which are in the WM dilated region, as follows:

(2) $$\begin{array}{c} \text{LesionMask}\left(\;x\;\right)=\left\{\begin{array}{cc} 1 & \text{FLAIR}\left(x\right)>\text{TH}\cap x\in \text{WM}\\ 0 & \text{otherwise}, \end{array}\right. \end{array}$$

where *x* is a voxel in the test image and TH is defined similar to [26]

(3) $$\begin{array}{c} \text{TH}=\mu_{GM}+\lambda \cdot \sigma_{GM}. \end{array}$$

*μ*_GM_ and *σ*_GM_ are the mean and standard deviation of the GM tissue in the FLAIR test image, respectively. The parameter *λ* is an empirical parameter, selected experimentally as 0.5. The extracted LesionMask, which includes the set of candidate lesion regions, contains around 20% of the brain voxels, but more than 95% of the lesions. This stage significantly reduces the number of voxels that need to be further analyzed for lesions. It was found in [26] to decrease false positives (FPs), almost without any degradation of the true positives (TPs), that is, less than 5% loss. The parameter *λ* was chosen as the maximum value that maintains 95% of the lesions in the candidate lesion regions. We optimized this parameter between 0 and 1 with increments of 0.1. Note that for *λ* = 0 (i.e., TH = *μ*_GM_) the results were almost identical.

### 2.5. Generation of an Initial Label Map

After the procedure described above, the voxels marked by the mask are further analyzed as lesion or nonlesion using a patch-based decision method. This patch-based segmentation strategy is based on the NLM estimator [6] that has been tested on a variety of tasks [1, 2, 26]. Likewise, in our work, given an augmented patch from a test image (combining several MR channels in the patch definition), similar patches are found in the labeled database described above.

Given input image *I* and the selected patch database *D*_*I*_′, the similar patch labels are combined to yield a lesion segmentation map, as follows.

#### 2.5.1. Patch Matching

For each voxel *x* in the test image, we extract a corresponding patch *P*_*x*_ centered at *x*. We then retrieve *k* similar patches *P*_*i*_^*D*^(*x*), *i* = {1,…, *k*}, from the database *D*_*I*_′. Each selected patch is weighted as follows:

(4) $$\begin{array}{c} w\left(\;x,P_{i}^{D}\;\right)=\exp\left(\;-\frac{d\left(P_{x},P_{i}^{D}\left(x\right)\right)}{\sigma^{2}}\;\right), \end{array}$$

where *d*(·, ·) is the square Euclidean distance and *σ* ^2^ is the maximum distance measure obtained for all the voxels *x* and for all the patches in the database *D*_*I*_′. Equations (6) and (7) below are alternative metric definitions for modalities balancing and spatial refinement, respectively.

#### 2.5.2. Vote Aggregation

To obtain a label for a test voxel *x*, a vote aggregation method is defined using all labeled patches extracted via the *k*-nearest neighbors (NN) patch matching procedure. The voxel *x* appears in *l* = 27 test patches centered on *x* and its neighboring voxels. For each test patch *P*_*x*_ we extract *k*-nearest neighbor labeled patches *P*_*i*_^*D*^(*x*)∣*i* = 1,…, *k* from the dataset *D*_*I*_′. Each retrieved similar patch, along with its labels {*l*_*i*_^*D*^(*x*)∣*i* = 1,…, *k*} and its weight, contributes a vote. Thus, for a given voxel *x* we have *l* · *k* votes that are averaged. The aggregation of votes results in a probabilistic lesion decision, as follows:

(5) $$\begin{array}{c} p\left(\;\text{lesion}\;|\;x\;\right)=\frac{\sum_{y\in P_{x}}^{}\sum_{i=1}^{k}w\left(y,P_{i}^{D}\left(y\right)\right)\cdot l_{i}^{D}\left(y,x\right)}{\sum_{y\in P_{x}}^{}\sum_{i=1}^{k}w\left(y,P_{i}^{D}\left(y\right)\right)}, \end{array}$$

where *y* goes over the 27 neighboring voxels of *x* and *l*_*i*_^*D*^(*y*, *x*) is the label that the training patch *P*_*i*_^*D*^(*y*) (which is similar to the patch centered at *y*) assigns to *x*. The final lesion label, *L*(*x*), for each voxel *x*, is defined as 1 if *p*(lesion∣*x*) > 0.5 and 0 otherwise. This content-based segmentation process results in an initial label map.

#### 2.5.3. Modalities Balancing

We examined the use of different weights for the three modalities, *M* = {T1, T2, FLAIR}, using multiplication by three constants, *C*_*M*_ = {*C*_T1_, *C*_T2_, *C*_FLAIR_}. This can be achieved by reformulation of the metric for the patch-based voting aggregation algorithm described above. The augmented patch (*P*_*x*_) is a concatenation of the T1 patch (*P*_*x*_^T1^), the T2 patch (*P*_*x*_^T2^), and the FLAIR patch (*P*_*x*_^FLAIR^); thus the balanced metric between a test patch *P*_*x*_ and a training patch *P*_*D*_ is defined as follows:

(6) $$\begin{array}{c} d_{I}\left(\;P_{x},P_{D}\;\right)=\underset{M}{\sum }C_{M}\cdot d\left(\;P_{x}^{M},P_{D}^{M}\;\right). \end{array}$$

The balanced metric has the opportunity to treat each channel of the MRI acquisition differently and, hence, give more weight to the more incriminating channel. This metric also enables examination of the patch source, that is, using one, two, or three modalities. For example, by using *C*_*M*_ = {*C*_T1_ = 1, *C*_T2_ = 0, *C*_FLAIR_ = 1} T2 is ignored.

### 2.6. Patch-Based Label Refinement

**Figure 4:**
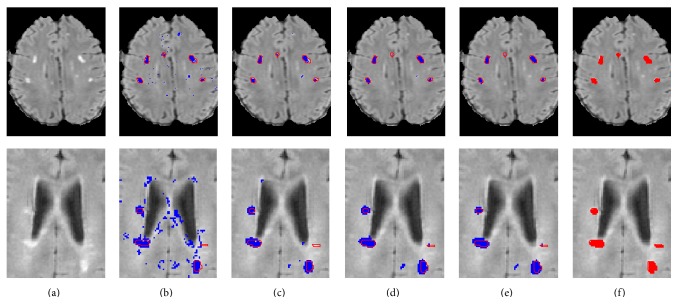
Two segmentation examples (top: UNC06, bottom: UNC02): (a) FLAIR image; (b) first iteration; (c–e) iterations 2, 3, and 5; (f) ground truth. Proposed method in blue; reference segmentation in red.

The above procedure treats each voxel independently and ignores spatial consistency constraints. For example, it may be the case that a single voxel is labeled as nonlesion even though all its neighboring voxels are labeled as lesions (see, e.g., the segmentation results shown in Figure 4(c)). In the following, we focus on incorporating spatial consistency into the lesion map result. As noted in [13], a segmentation method that relies solely on voxel intensity is unlikely to produce sufficient results. As such, many attempts have been made to incorporate spatial or anatomical information within the segmentation process [8, 42]. In other patch-based segmentation algorithms [1, 2] a search volume is defined around the voxel under study. However, this cannot be applied in a MS lesion task given the lack of clear anatomical location, in addition to the potential weakness of reliance on good affine registration in the presence of lesions.

In Wang et al. (2013) [40] an alternative framework that combines the use of *k*-NN ball trees and a spatial weight label fusion scheme to search for patches in large regional areas was suggested. This Spatially Aware Patch-Based Segmentation (SAPS) is designed to overcome the problem of limited search windows and combine spatial information by using the anatomical location of the patch. Similarly, in Wang et al. (2014) [42] an iterative refinement step using a sparse representation was implemented to correct the anatomical errors introduced in the segmentation. Our goal is to use the label information to enforce a consistency of the decision. The term* spatial consistency* is adapted from Freeman et al. [5] where the authors proposed a Markov random field solution for spatial consistency. They introduced a one-pass algorithm that used the neighbors relation in a similar way to the one reflected in the MRF pixel grid.

In our method we enforce spatial consistency using a new metric for the patch-based voting aggregation algorithm described above. The new metric uses the current label map in addition to the intensity information. A weighting parameter, *α*, is used to determine the relative importance of the intensity similarity and the spatial consistency constraint. This new metric is used in the patch matching step and in the vote weighing step. The metric between a test patch *P*_*x*_ and a training patch *P*_*D*_ is defined as follows:

(7) $$\begin{array}{c} d\left(\;P_{x},P_{D}\;\right)=d_{I}\left(\;P_{x},P_{D}\;\right)+\alpha \cdot d_{L}\left(\;P_{x},P_{D}\;\right), \end{array}$$

where *d*_*I*_(·, ·) is the intensity-based metric described above and *d*_*L*_(*P*_*x*_, *P*_*D*_) is the square Euclidean distance between the current labels of the patch *P*_*x*_ and the labels of training patch *P*_*D*_. A larger weight parameter *α* tends to favor the current labeling decision of the algorithm. We use the following definition for the weight:

(8) $$\begin{array}{c} \alpha_{t}=\alpha_{0}*\left(\;t-1\;\right) \end{array}$$

with constant *α*_0_ ensuring that the intensity metric and label metric are on the same scale and *t* is the iteration number. In the first iteration, *α* = 0; thus we start with the patch-based method as described in Section 2.3. As *α* is increased from one iteration to the next, it gives more weight to labels of the nearest-neighbor training patches in making the current label decision. A large *α* ensures that the labels of the nearest-neighbor training patches coincide with the current label decision and therefore the algorithm converges. We have found that in practice there is no need for more than 4-5 iterations until convergence.

Our method imposes a global consistency constraint between the labels of neighboring voxels in an efficient way. For each patch we find the most similar patch based on intensity while also taking into account spatial compatibility with the neighboring voxels. The initial patch-based segmentation results are sensitive to small hyperintense regions that are caused by noise and inhomogeneities. These regions contain patches that are similar to lesion patches. The majority of these voxels are correctly labeled as nonlesions during the refinement stage.  Algorithm 1 summarizes the proposed segmentation algorithm.

### 2.7. Implementation Details

The proposed method was implemented in MATLAB 8.0 using C/MEX code and the experiments were conducted using an Intel Core i7-3770 processor at 3.4 GHz with 8 GB RAM. The preprocessing steps were carried out with using BrainSuite13 [34], 3DSlicer [43], and MATLAB. The average overall runtime for a single image is less than 4 minutes.

## 3. Results

This section reports on the segmentation results and compares the patch-based approach to other top-ranked methods [14–16].

### 3.1. Datasets

We evaluated our framework using clinical public data provided by the MS lesion segmentation challenge which was introduced at the MICCAI MS lesion segmentation workshop 2008 [22]. This is the largest dataset publicly available, and most recent works in the field use this dataset as it provides a benchmark for algorithm comparisons. The MS lesion grand challenge offers two datasets: a labeled dataset (originally designed for training) and an unlabeled dataset (used for testing). We will hereon term them the public set and private set, respectively. The public set (train set) contains 20 cases, 10 from the University of North Carolina (UNC) and 10 from the Children's Hospital Boston (CHB) datasets. The private dataset (testing set) contains 15 cases from CHB and 10 from UNC. For the private dataset two lesion markings are available, segmented by UNC and CHB rater. The dataset shows high variability in intensity contrast, image noise, and bias field. The dataset contains highly heterogeneous cases and can thus be considered as a realistic test case. In all cases, the data resolution is 0.5 mm^3^ isotropic in three different modalities (T1w, T2w, and FLAIR). All data were rigidly registered to a common reference frame and resliced to isotropic voxel spacing using b-spline based interpolation by the challenge organizers. Full documentation is available in [22].

### 3.2. Evaluation

#### 3.2.1. MICCAI2008 Public Dataset (Training Set)

Our procedure was evaluated in a leave-one-out framework for each medical center (CHB and UNC). We computed true positives (TPs), true negatives (TNs), false positives (FPs), and false negatives (FNs) and used the following validation measures: True Positive Rate (TPR) defined as TP/(TP + FN), Positive Predictive Value (PPV) defined as TP/(TP + FP), and Dice Similarity Coefficient (DSC) defined as (2TP)/(FP + FN + 2TP). The measures were computed using the expert label map provided in the dataset. The scores appear in Table 1 along with a comparison to three state-of-the-art works using the same dataset. The three selected works are Souplet et al. [15], the winner of the MICCAI MS lesion grand challenge [22]; Weiss et al. [16], the latest work published using this database; and Geremia et al. [14], one of the outstanding supervised methods published. Note that DSC measures were not provided by the authors in the first two methods.

The proposed method achieved a mean TPR of 40%, a mean PPV of 29%, and a mean DSC of 31%. These results are comparable with the other state-of-the-art algorithms using the same data. Two successful segmentation examples are shown in Figure 4. The input image is presented in Figure 4(a) and the final segmentation map is shown in Figure 4(e). The similarity to the reference segmentation, shown in Figure 4(f), is evident. There was a substantial decrease in FPs, as part of the algorithm iterative refinement procedure, in both cases. The reduction in FPs is more significant in the second iteration. In addition, observe an increased number of border voxels being classified as lesions in iterations 3 to 5. Thus overall there was a significant decrease in the quantity of the FPs and an increase in all the measured indices. The result analysis in this section is based on the public dataset.

#### 3.2.2. MICCAI2008 Private Dataset (Testing Set)

Quantitative evaluation was also carried out on the private dataset using a set of known metrics defined in [22]. In order to allow quantitative comparison between methods and to human export the organizers define average score. Each metric is related to the result that could be expected if an independent human observer would perform the segmentation manually. Thus 100 points mean a perfect result (the best value that could be obtained for a metric) and a predefined amount (see [22] for details) of 90 for a score that is typical for an independent human observer. The algorithm results on the MICCAI2008 private dataset, as provided by the MS-lesion challenge organizers, are available in the challenge website (http://www.ia.unc.edu/MSseg). The proposed method achieved an average score of 72%, where the average scores on UNC and CHB databases were 68% and 74%, respectively. Generally, a significant difference is reflected when comparing the method results on the UNC (68%) and CHB (74%) datasets. It is hard to understand the specific reason for that difference in the absence of the GT. We believe that this difference could be explained by changes in the MRI acquisition parameters which the normalization steps failed to bridge.

### 3.3. Computational Time

Due to the high dimension of the search space, finding the absolute best match would be computationally prohibitive. Instead, we used FLANN (Fast Library for Approximate Nearest Neighbors) [44, 45]. FLANN is a library for performing fast approximate nearest neighbor searches in high dimensional spaces. It contains a collection of algorithms found to work best for nearest neighbor searches and a system for automatically choosing the best algorithm and optimum parameters depending on the dataset. Using FLANN, each NN iteration takes less than 1 minute (≈40 sec) and results in a total time of less than 4 minutes. Note that when we compared the approximated result to an accurate result achieved by brute force NN implementation, the results were similar, without a significant disparity. Computational times in other methods were not specified in a way that enables comparison. In Souplet et al. [15], an example of execution time on one case was given, and the computational time reported (without the preprocessing) was 34 min. Weiss et al. [16] reported 5 min, which is similar to the patch-based method. Geremia et al. [14] did not report the computational time for a single test case, but rather a significant training time of 8 hours to train the random forest classifier they used. All frameworks use similar preprocessing steps; thus the comparison of preprocessing time is irrelevant.

### 3.4. Visualization of the Lesion Data in Intensity Space

**Figure 5:**
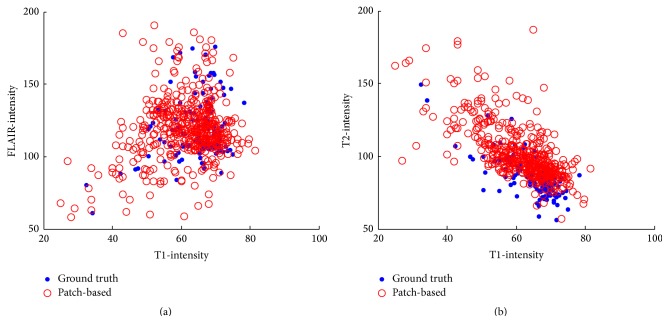
Gray level feature space for the lesions in all images. (a) FLAIR and T1 feature space; (b) T2 and T1 feature space. The blue dots represent the mean intensity of the lesions as marked by the expert. The red dots represent the mean intensity of the lesions as detected by the algorithm.

The driving force behind the patch-based approach is the intensity similarity between the patches. We compared the mean intensity of the lesions detected by the algorithm, to those marked by the expert.  Figure 5 shows the mean intensity of the lesion across the multimodal images. Generally, the fit between the lesion dots is high, suggesting that the intensity range of our method is compatible with the ground truth. Each sample in Figure 5 represents one lesion; some of the lesions in the ground truth (the full blue dots) appear to be significant outliers, for example, the two lesions with the lowest FLAIR and T1 intensities and the highest T2-intensity (FLAIR(T1) graph: upper left corner, T2(T1) graph: bottom left corner). The sources of these lesions are patients UNC01 and CHB05. Furthermore, this figure emphasizes the importance of the subject selection step for outlier handling.

### 3.5. Influence of the Patch Database Definition

One of the greatest challenges in the system is defining the patch database. It is clear that if the database characterization contains too few patches or represents the brain tissue and the lesions poorly this can lead to misclassification. FP errors are instances of retrieved patches which are similar to lesions but originate from healthy tissue, and therefore their labels lead to misclassification. We also tried to use only the patches in the ROI marked as example in Figure 2 (Section 2.3) and the results were slightly worse. Furthermore, the WM segmentation mask may contain classification errors or adjacent tissues which may cause the need to decide whether patches from other tissues are lesion or not.

**Figure 6:**
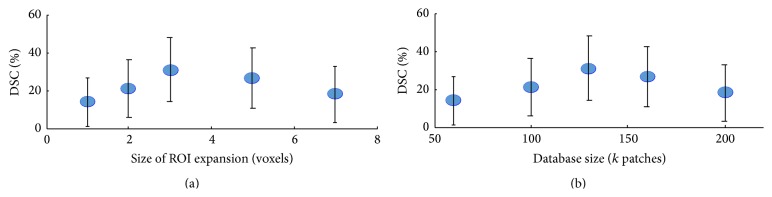
(a) DSC (%) as function of the number of voxels expansion per ROI. Recall that we expand the borders of a lesion bounding box by a few voxels in order to capture the surrounding tissue as well. The *x*-axis represents the number of voxels in this expansion. (b) DSC (%) as function of the number of patches in the database, which was obtained from the ROI expansion size.

In classical machine learning systems, the training data and the testing data are usually identical in terms of their properties. That is, to classify all the brain voxels, it makes sense to train all of them. However, creating a huge database, containing millions of patches would cause the classifier to favor the healthy class. Therefore, we chose to build the database with patches extracted from the lesion regions. The parameter that determines the amount of patches that are proximal to the lesions is the size of the bounding box around the lesion. As the bounding box is larger it will contain more healthy patches and the database size will increase.  Figure 6 shows the influence of this parameter on the results (DSC). The results in Figure 6(a) support our selection of a bounding box expansion of 3 voxels. From Figure 6(b) we see that the best DSC result was obtained for a patch number between 100,000 and 150,000 patches.

### 3.6. Influence of Number of Nearest Patches (*k*) and Subject Selection (*N*)

**Figure 7:**
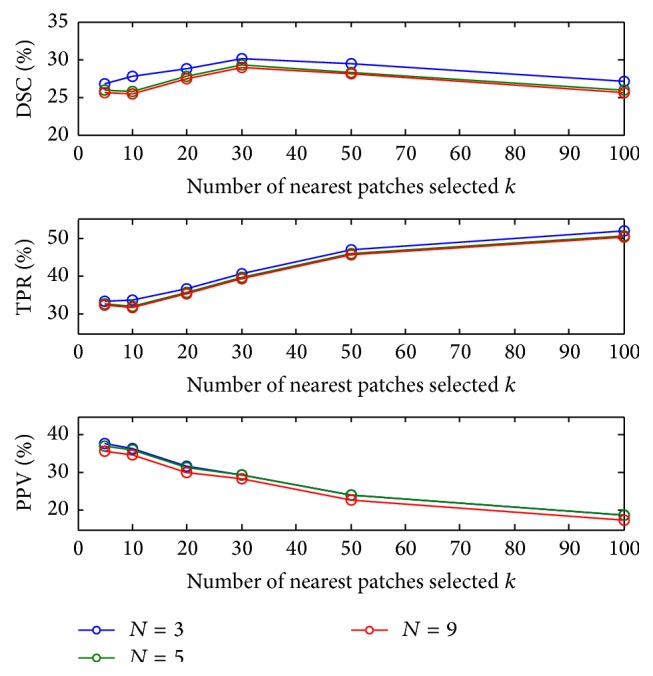
DSC (%) for a range of number of nearest patches *k* with different number of selected subjects *N*.

Figure 7 depicts performance change as a function of the number of nearest patches *k* and for different number of selected subject *N*. It shows that TPR increases and PPV decreases as *k* increases, which causes the DSC to remain almost unchanged. We chose an optimal working point, where the PPV and TPR are similar; that is, *k* = 30.

Figure 7 shows that the optimal *k* value is similar for different *N* values. A slight improvement was observed for *N* = 5. When the number of training images is very large, images that are not very similar (large KL divergence in the subject selection stage) are also used, and thus the patch matching is biased. Conversely, for *N* = 1 the patch selection is too small, and thus for some of the patches similar patches are not found.

Generally, we found that the True Positive Rate increases as *k* increases; this could be explained by the fact that in (4) we calculate the weights for each patch vote. For dissimilar patches this weight is practically zero; thus for *k* > 100 the results are with small change. In practice we selected *k* = 30 because when using more patches, the algorithm tends to classify the patches as lesions. This can be explained by the balanced labeled database we used. As explained in Section 2.3.1, the selection of the patches represented the lesion and nonlesion in a roughly balanced way; thus when *k* > 30 the algorithm finds patches that are quite similar to the target patch with high probability to be lesion. Moreover, we found that the patches retrieved after the 30th patch are different one from the other mainly in the noise level; this causes the classification probability to be as in the database, roughly balanced, and thus decreases the overall performance (while the TPR is increasing).

### 3.7. Influence of the Spatial Refinement

**Figure 8:**
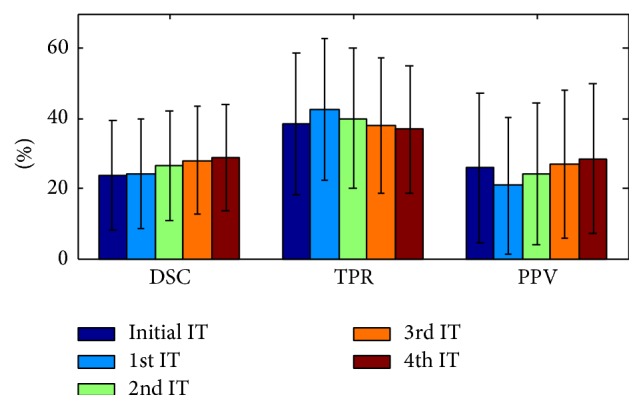
TPR/PPV/DSC (%) with respect to iteration (IT) number (using *k* = 30 and *N* = 5). The first iteration is the initialization step and the remainder are label refinement steps.

Figure 8 shows the effect of the iterative refinement step. An increase is seen in the DSC from one iteration to the next. This increase was observed consistently for all subjects. A mean DSC value of 21% was achieved before the patch-based label refinement process; a mean DSC of 25% was obtained after two iterations and in the last iteration we achieved a mean DSC of 31% which shows a substantial improvement.

The weighting for the spatial information is determined by *α*_0_. Specifically, we selected a value of 20, which results in parameter *α* to range between 0 and 80 (from the initial iteration to the fifth). The value of *α* in the fifth iteration is similar to the average intensity of a lesion (across all MR channels).

### 3.8. Influence of Modality Combinations

**Figure 9:**
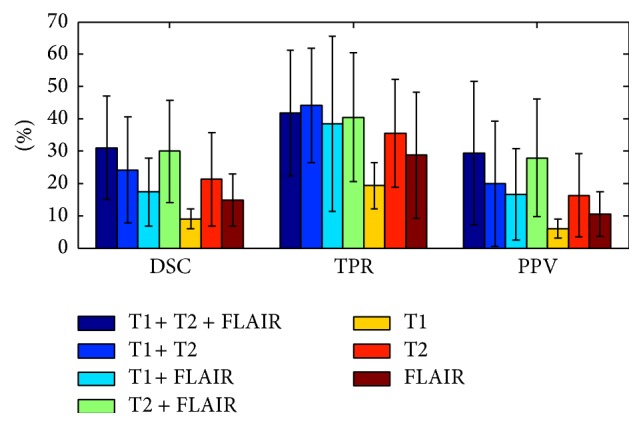
Segmentation performance with different modality combinations (using *k* = 30 and *N* = 5). The results reported in Section 3.2 and Table 1 are with all three modalities.

In this work, augmented patches are used; that is, we concatenate the patches across the different MRI modalities. Using the augmented patches in this way ensures that all three modalities are approximately equally treated. Using the Euclidean distance, which is sensitive to the intensity of the patch, may in fact result in the fact that the brightest modality (FLAIR) has the greatest effect. Still, the effect of the differences in intensity is relatively random, making it legitimate to assume that the overall effect on patch matching is negligible. The segmentation performance was evaluated for various combinations of modalities.  Figure 9 shows DSC, TPR, and PPV values for three single-modality, three double-modality, and one multimodal patch sources. When using a single modality, T2 resulted in the best performance. Combining across several modalities reduced the FPs and increased the TPs. On the entire dataset, the combination T1w + T2w + FLAIR performed statistically better than all other combinations (aside from T1w + T2w in the TPR measure).

**Figure 10:**
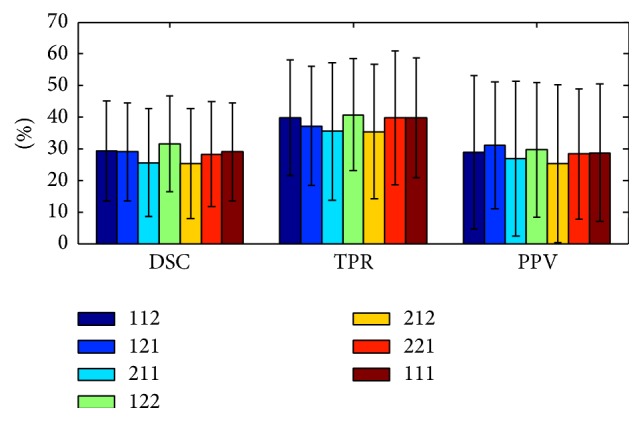
Segmentation performance with different modality weights (using *k* = 30 and *N* = 5). The numbers in the legend from left to right are the modality weights *C*_T1_,  *C*_T2_, and *C*_FLAIR_ in the metric, respectively. (The results reported in Section 3.2 and Table 1 are with *C*_T1_ = *C*_T2_ = *C*_FLAIR_ = 1.)

Figure 10 shows DSC, TPR, and PPV values for five different modality weights *C*_*M*_ = {*C*_T1_, *C*_T2_, *C*_FLAIR_}. In this experiment, we did not perform an accurate optimization of the three weight parameters. Our goal was to identify the influence of the parameter set and its possible future use in patch-based segmentation approaches. Slightly better results were achieved using *C*_T2_ = 2 and *C*_FLAIR_ = 2. This result is compatible with the fact that the main characteristic of a WM lesion is that its intensity is brighter than its surroundings on T2w and FLAIR.

## 4. Discussion and Conclusion

In this paper, we proposed a novel patch-based method for detection and segmentation of MS lesions in brain MRI by utilizing multimodal spatial information. The segmentation is first obtained based on intensity patch similarity and then further iteratively refined with the spatial label information. The proposed framework segments the MS image without requiring registration or an atlas. This makes the framework robust to registration errors which are likely to occur if there is a high degree of anatomical variability.

The relationships between patches can be modeled as a Markov Network (or random field) [46]. We let the test brain image patches be observation nodes and selected the *k* closest examples to each input patch as the different states of the hidden nodes that we sought to estimate. In [5] a good approximate solution for this relationship was obtained by running a belief propagation. These authors also introduced a new one-pass algorithm that computes the patch compatibilities for previously selected neighboring patches. In our algorithm we use this idea in order to take the relationships between the labels of neighboring patches into account.

A few main characteristics of the presented approach include the following: (1) The patch-based segmentation method is nonparametric. We do not assume any intensity models and rely only on intensity information. In other parametric models the amount of lesion load needs to be determined a priori. (2) We integrate the spatial information through the patch-based metric by incorporating the label term. The spatial refinement step adds both the neighborhood relations and introduces spatial consistency. (3) The proposed framework is supervised and thus aims to represent the lesion and nonlesion tissues using labeled data. In other unsupervised methods, finding outliers in the model is the guiding principle.

In this paper, we compared the proposed method against three other methods. In Souplet et al. [15], the authors showed that a global threshold on the FLAIR MR sequence, inferred using EM brain tissue classification, suffices to detect most MS lesions. The final segmentation is then constrained to appear in the white matter by applying morphological operations. The method reported here achieves better accuracy than the Souplet et al. method. In Geremia et al. [14], the authors proposed a discriminative random decision forest framework to provide a voxel-wise probabilistic classification of the volume. The method uses multichannel MR intensities, knowledge about tissue classes, and long-range spatial context to discriminate lesions from background. A symmetry feature is introduced to account for the fact that some MS lesions tend to develop in an asymmetric way.

When compared to this method, our findings are mixed. This may be due to the fact that in the current work we are using intensity only features. This representation is sensitive to normalization. In their work Geremia et al. indicated that sophisticated features such as context-rich and symmetric features reduce the strong normalization preprocessing requirements. We therefore believe that combining some of the context-rich and symmetry features may enable us to reach better results. In Weiss et al. [16], an unsupervised approach addressing the problem with dictionary learning and sparse coding was used. This method is the most recent published work addressing the MS lesion segmentation task using the MICCAI08 datasets.

It is interesting to note the low results for subject UNC01, for both our algorithm and Souplet et al. [15] and Geremia et al. [14] algorithm. This specific case contains two lesions, where the first is relatively small and the second is very similar to the CSF tissue and touches it (Figure 5 shows the significant intensity outlier). In addition, when comparing the average intensity of the second lesion in all three channels, it appears as a significant outlier.

One of the major drawbacks of MRI is the lack of standard and quantifiable interpretation of image intensities. Unlike other modalities, such as X-ray computerized tomography, MR images of the same patient taken on the same scanner at different times may appear different from each other due to a variety of scanner-dependent variations and therefore the absolute intensity values do not have a fixed meaning [32]. The key step in overcoming this drawback is a normalization step during preprocessing (Section 2.2); this enables intensity-based patch comparisons. We use state-of-the-art intensity normalization schemes. Still, we have found high sensitivity to any intensity differences. For this reason we added a subject selection stage. Only the more similar subjects have a patch-comparison conducted. Out of the 20 subject MR images in the datasets, a few of them (e.g., CHB2 and CHB5) differ markedly. The impact was considerable on these subject's segmentation results and in fact went from total segmentation failure to reasonable results. In the patch selection stage (Section 2.3) a fixed number of patches were selected (150 K). In our experimentations we have found that this fixed number of patches is satisfactory for any training data size, as long as the patch database formed contains sufficient diversity. The fixed number of patches selected provides invariance to data size and any scalability concerns.

In future research we plan to explore various additional representations for the patches, such as using the Histogram of Oriented Gradients (HoG [47]). One possible explanation for the lower results we achieved on the private dataset is the difficulty of the subject selection step to find sufficiently good matches for the test cases. In other words the advantage of comparing patches from a variety of brains and tissues caused misclassification of lesions. Evidence for that is the relative high score on the volume difference as compared with the FPR and TPR scores.

This framework could be coupled with dictionary learning, as well as additional pre- and postprocessing to improve performance. In future work we aim to improve the results using better database characterization and advanced metric learning. Evaluation of our algorithm on larger datasets from varying input sources will allow us to test the robustness of the algorithm to noise and other variance. We are now working on a system for screening of highly noisy patches, or patches which are extracted from fundamentally different brains. We believe that better learning of the database will lead to a considerable improvement in the results.

To conclude, we presented a novel framework for patch-based segmentation that integrates intensity information with a patch-based label refinement. The method does not call for nonlinear alignment of the training images onto the space of the testing image. Many other works use this step, which is a major source of error and is computationally expensive. The database concept, as the novel refinement step, can be easily applied in variety of patch-based segmentation frameworks. Although the patch-based algorithm is based on a *k*-NN search, a good approximation for the search was found to result in less than 5 min. The total segmentation time (not including preprocessing) makes the method one of the fastest proposed for the MS lesion task. The method proposed is a general one and as we believe can be generalized to other small lesion tasks such as liver metastasis and chest X-ray pathologies.

## Figures and Tables

**Algorithm 1:**
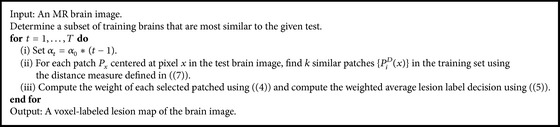
The lesion detection and segmentation algorithm.

**Table 1:** Comparison of current method to three state-of-the-art methods on clinical brain image data from the public part of MICCAI 08 challenge [22]. TPR, PPV, and DSC are in %, where 100 is perfect segmentation.

Patient	Souplet et al. [15]		Geremia et al. [14]		Weiss et al. [16]			Current method		
	TPR	PPV	TPR	PPV	TPR	PPV	DSC	TPR	PPV	DSC
UNC01	1	1	2	1	33	29	31	0	0	0
UNC02	37	39	48	36	54	51	53	67	34	45
UNC03	12	16	24	35	64	27	38	57	25	35
UNC04	38	54	54	38	40	51	45	65	17	27
UNC05	38	8	56	19	25	10	16	36	9	14
UNC06	57	9	15	8	13	55	20	37	69	48
UNC07	27	18	76	16	44	23	30	51	48	49
UNC08	27	20	52	32	43	13	20	24	11	15
UNC09	16	43	67	36	69	6	11	29	35	32
UNC10	22	28	53	34	43	23	30	44	45	45

Average	28	24	45	26	43	29	29	41	29	31

CHB01	22	41	49	64	60	58	59	40	33	36
CHB02	18	29	44	63	27	45	34	41	11	17
CHB03	17	21	22	57	24	56	34	47	19	27
CHB04	12	55	31	78	27	66	38	37	7	12
CHB05	22	42	40	52	29	33	31	58	25	35
CHB06	13	46	32	52	10	36	16	43	38	41
CHB07	13	39	40	54	14	48	22	34	50	41
CHB08	13	55	46	65	21	73	32	48	52	50
CHB09	3	18	23	28	5	22	8	31	23	26
CHB10	5	18	23	39	15	12	13	13	28	18

Average	14	36	35	55	23	45	29	39	29	30
